# Supplementary material for: Quantum Biochemistry Screening and In Vitro Evaluation of Leishmania Metalloproteinase Inhibitors
Source: Int J Mol Sci. 2022 Aug 2;23(15):8553. doi: 10.3390/ijms23158553 (PMC9368959; doi:10.3390/ijms23158553)
Supplement: Supplementary file 1 [file ijms-23-08553-s001.zip › ijms-1785126-supplementary.pdf]

Supplementary Materials

Figures

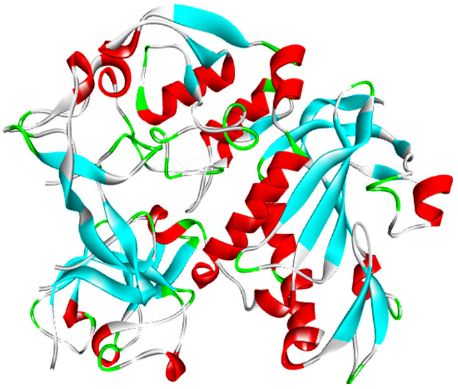

Figure S1. Leishmanolysin crystal structure (1LML). Visualization of the secondary structure of the Leishmanolysin or glycoprotein of 63 kDa (gp63) from *Leishmania major* by Discovery Studio Visualizer Software.

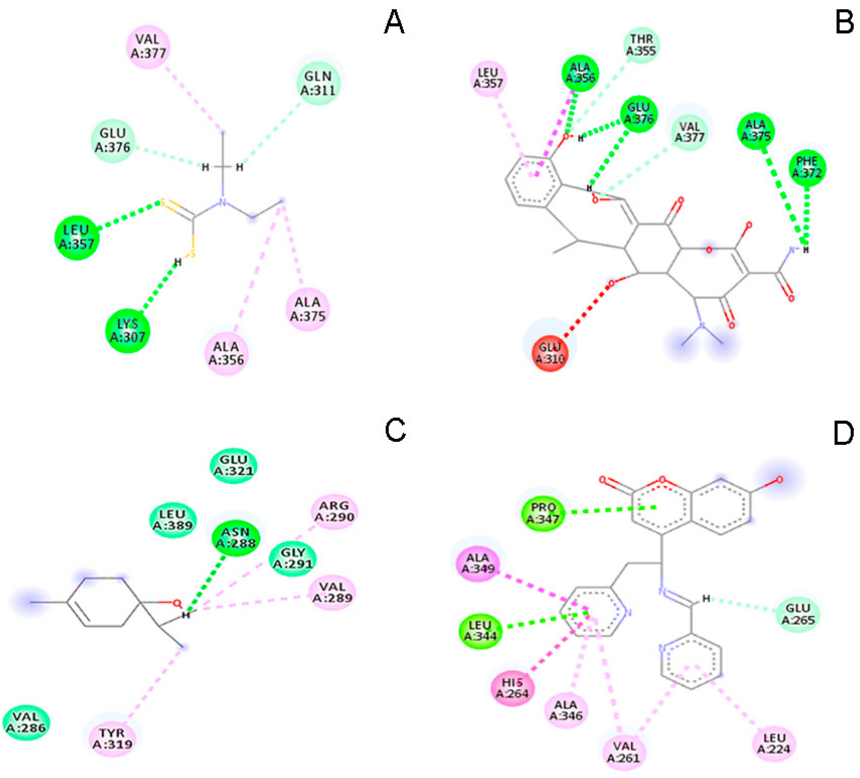

Figure S2. 2D visualization of the (**A**) DETC, (**B**) Doxycycline, (**C**) Terpinen-4-ol, and (**D**) L1 ligands and the most interacting gp63 amino acid residues using Discovery Studio Software.

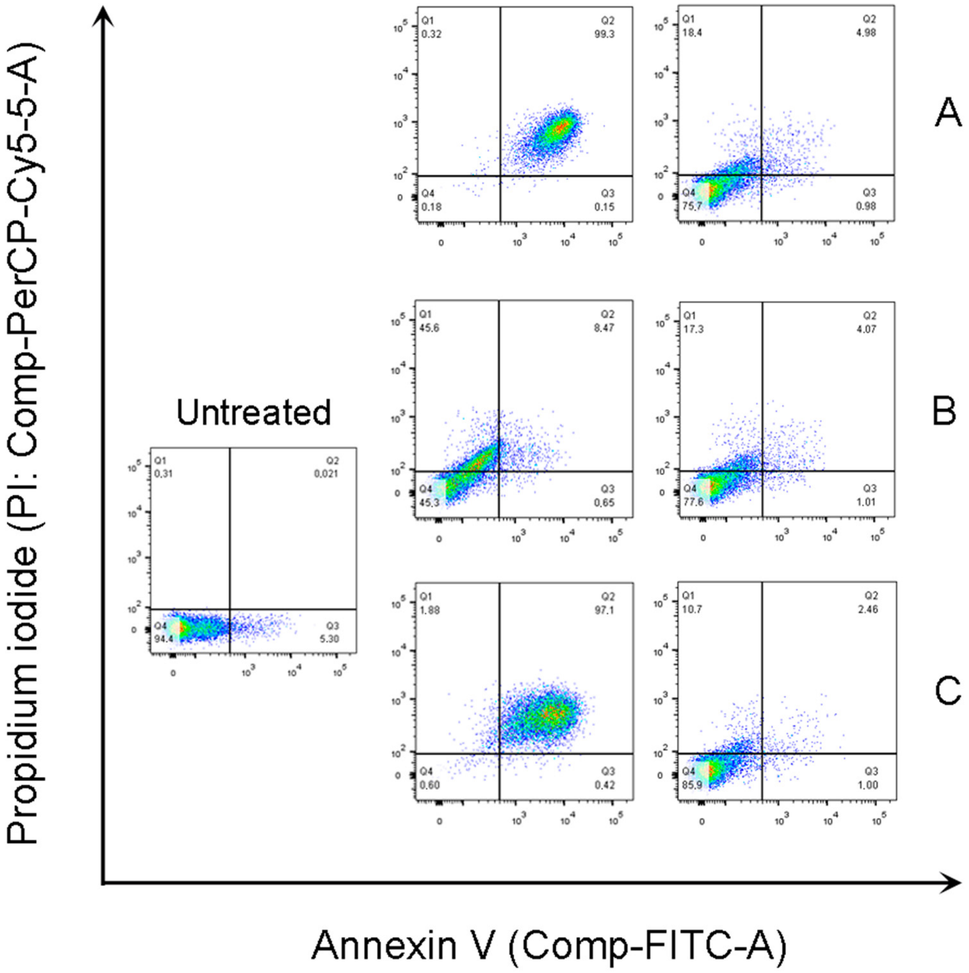

Figure S3. Flow cytometry analysis of *L. amazonensis* promastigote forms after 24-hour incubation with Amphotericin B [(**A**): left - 10,8 μM and right 0.108 μM]; DOXY [(**B**): left - 225 μM and right 22,5 μM] and DETC [(**C**): left - 443 μM and right 4.43 μM]. The assay tested Ic50 values and 100 times the value. Untreated promastigote forms were used as a negative control.

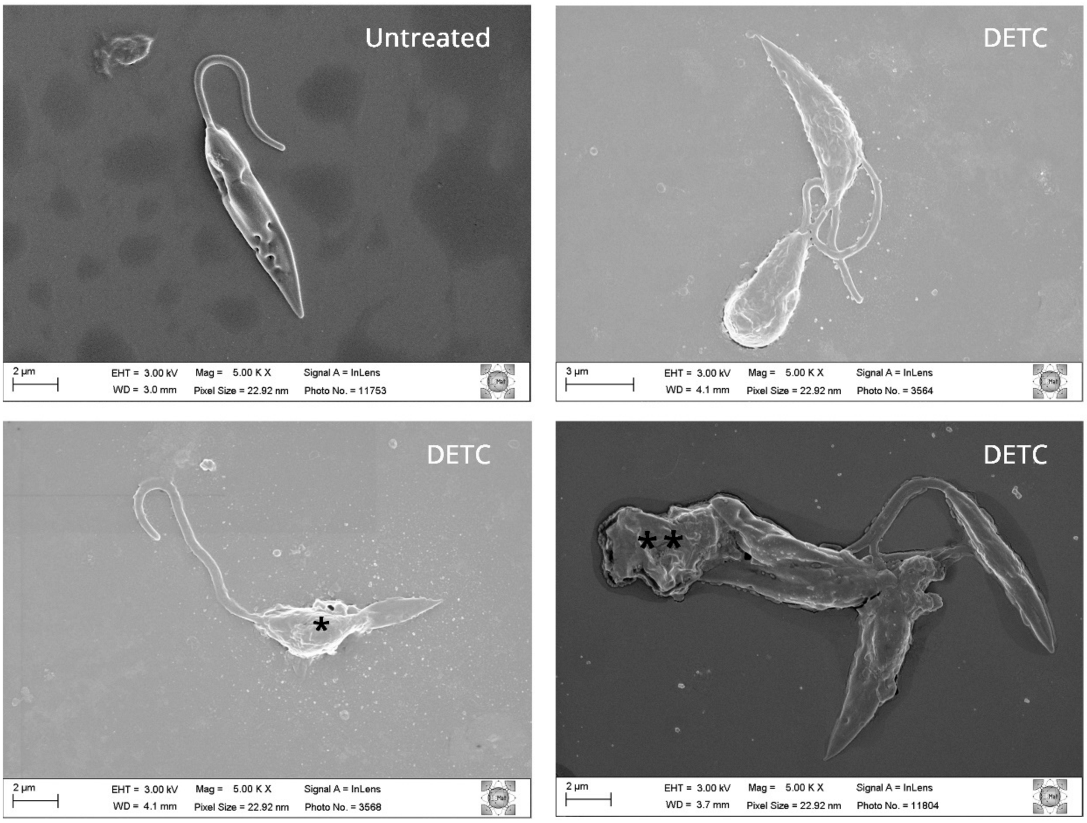

Figure S4. Effect of DETC on *Leishmania amazonensis* promastigote. Untreated parasites used as control. Promastigote forms treated for 24 hours with DETC IC_{50} concentration were observed by scanning electron microscopy and images were acquired. Treated parasites evidence membrane irregularities, destruction of cellular morphology, leakage of cellular content and cell retraction.

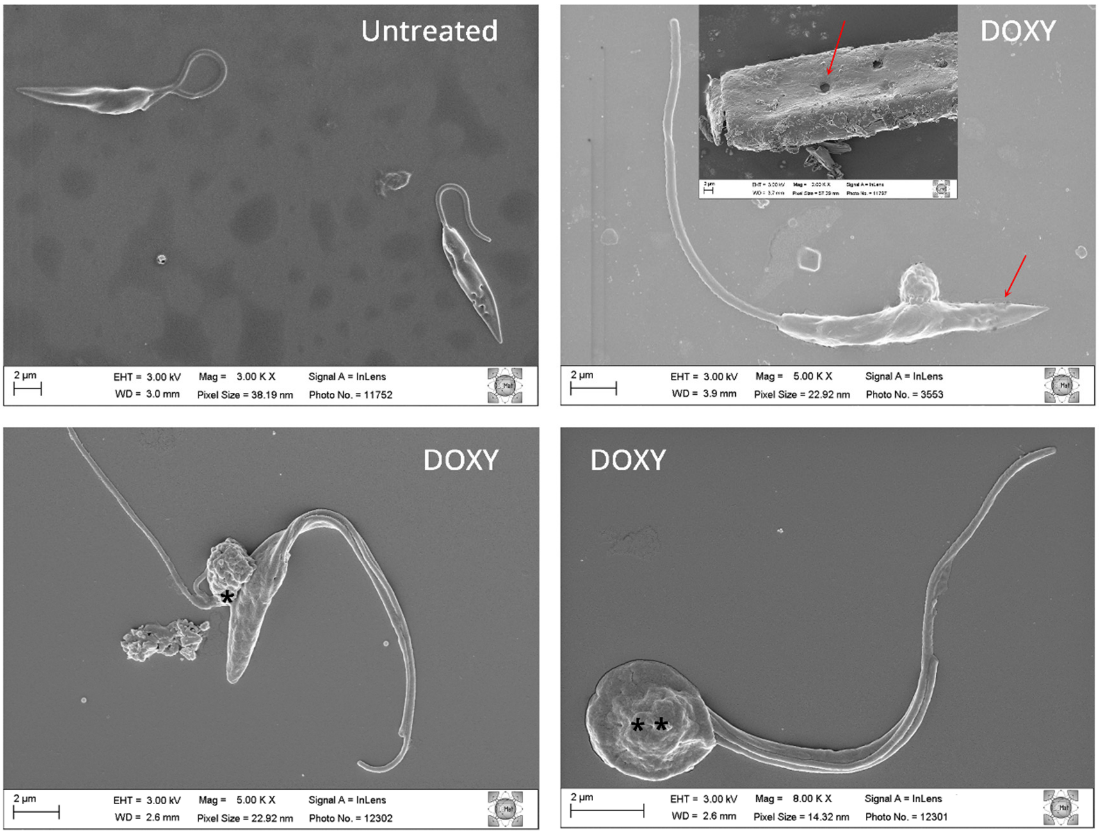

Figure S5. Effect of Doxycycline (DOXY) on *Leishmania amazonensis* promastigote. Untreated parasites used as control. Promastigote forms treated for 24 hours with DETC IC_{50} concentration were observed by scanning electron microscopy and images were acquired. Treated parasites evidence membrane irregularities, destruction of cellular morphology and flagella, membrane pores (Red row), and cell retraction (black asterisk).

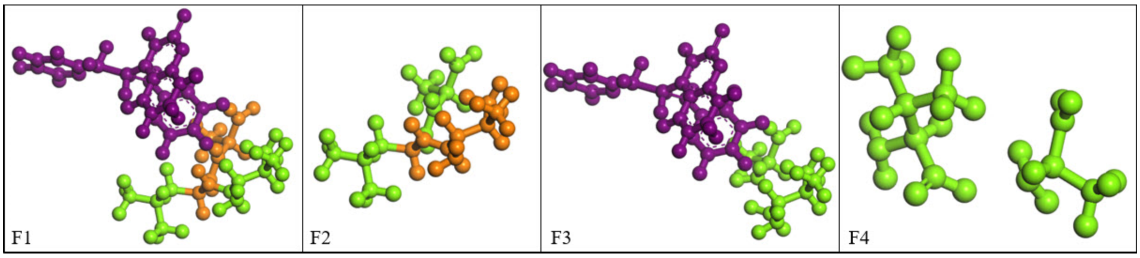

Figure S6: Schematic representation of molecular fractionation method with conjugated caps (MFCC) illustrated by established fragments: **F1 - F2 - F3 + F4**.

Tables

Table S1. Interaction of DETC and gp63 estimated by the AutoDock4 program.

**Complex**	**EE**	**FIE**
1	−3.21	−4.60
2	−3.1	−4.34
3	−3.36	−4.55
4	−3.41	−4.60
5	−3.28	−4.48
6	−4.45	−5.64
7	−3.53	−4.72
8	−2.98	−4.17
9	−3.40	−4.59
10	−3.28	−4.48

Estimated free energy (**EE**) and final intermolecular energy (**FIE**) in Kcal/mol

Table S2. Interaction of Doxycycline (DOXI) and gp63 estimated by the AutoDock4 program.

Complex	EE	FIE
1	−3.95	−6.04
2	−4.71	−6.80
3	−5.15	−7.24
4	−4.49	−6.58
5	−4.52	−6.61
6	-4.60	−6.68
7	−5.46	−7.54
8	−4.80	−6.89
9	−4.13	−6.22
10	−4.79	−6.88

Estimated free energy (**EE**) and final intermolecular energy (**FIE**) in Kcal/mol

Table S3. Interaction of Terpen-Ol-4 (TERP4) and gp63 estimated by the AutoDock4 program.

Complex	EE	FIE
1	−5.04	−5.64
2	−4.13	−4.72
3	−5.00	−5.60
4	−4.26	−4.86
5	−4.19	−4.79
6	−4.10	−4.70
7	−4.27	−4.87
8	−4.42	−5.01
9	−4.41	−5.01
10	−4.12	−4.72

Estimated free energy (**EE**) and final intermolecular energy (**FIE**) in Kcal/mol

Table S4. Interaction of L1 and gp63 was estimated by the AutoDock4 program.

Complex	EE	FIE
1	−5.56	−7.35
2	−5.90	−7.69
3	−6.38	−8.17
4	−5.80	−7.59
5	−6.97	−8.76
6	−6.11	−7.90
7	−7.97	−9.76
8	−8.07	−9.86
9	−6.20	−7.99
10	−6.93	−8.72

Estimated free energy (**EE**) and final intermolecular energy (**FIE**) in Kcal/mol
